# The First Annual Announcement of Hahnemann Medical College of San Francisco

**Published:** 1884-07

**Authors:** 


### The First Annual Announcement of the Hahnemann Medical College of San Francisco.

This college opens with bright prospects and a good faculty; it will be the centre of *homœopathic* education for the Pacific Coast.

